# Application of advanced diffusion MRI based tractometry of the visual pathway in glaucoma: a systematic review

**DOI:** 10.3389/fnins.2025.1577991

**Published:** 2025-05-21

**Authors:** Loxlan W. Kasa, Stephanie Donovan, Eryn Kwon, Samantha Holdsworth, William Schierding, Helen Danesh-Meyer

**Affiliations:** ^1^Mātai Medical Research Institute, Tairāwhiti Gisborne, New Zealand; ^2^Department of Ophthalmology, The University of Auckland, Auckland, New Zealand; ^3^Vision Research Foundation, Auckland, New Zealand; ^4^Centre for Brain Research, Faculty of Medical and Health Sciences, The University of Auckland, Auckland, New Zealand; ^5^Auckland Bioengineering Institute, Auckland, New Zealand; ^6^Eye Institute, Auckland, New Zealand

**Keywords:** diffusion kurtosis imaging, neurite orientation dispersion and density imaging, fixel-based analysis, tractometry, glaucoma, visual pathway, diffusion tensor imaging

## Abstract

**Background:**

Glaucoma is a leading cause of blindness globally, with emerging research suggesting that glaucoma-related degeneration may affect the visual pathway. Recent advancements in magnetic resonance imaging (MRI) offer promising non-invasive methods for evaluating glaucoma, including advanced diffusion MRI (dMRI) and computational techniques. One such technique is tractometry, which quantifies white matter (WM) microstructural properties. While the application of tractometry in glaucomatous patients is developing, several key studies have explored structural changes in the brain, particularly within the visual pathways, in individuals with glaucoma. This systematic review comprehensively evaluates the application of tractometry using advanced dMRI models and techniques to quantify WM in the visual pathway of individuals with glaucoma.

**Methods:**

PubMed-Medline and PubMed-Central were screened for articles published until April 11th, 2024. The studies based on patient cohorts affected by primary open-angle glaucoma (POAG), primary angle closure glaucoma (PACG), and normal tension glaucoma (NTG) with the following dMRI techniques and tract-specific analysis approach were included in this review: diffusion tensor imaging (DTI), diffusion kurtosis imaging (DKI), neurite orientation dispersion and density imaging (NODDI), fixel-based analysis (FBA), and dMRI tractometry.

**Results:**

The selected seven studies incorporate tractometry and advanced diffusion models and techniques (DKI, NODDI and FBA), including DTI. Each study investigated microstructural changes along the visual pathway of glaucomatous patients, finding glaucomatous WM degeneration in the optic nerve (ON), optic tract (OT), and optic radiation (OR), as well as significantly altered WM connections between the brain's visual cortex and non-visual areas. Additionally, tractometric findings correlated with clinical measures of glaucoma, such as intraocular pressure, visual field loss, and retinal nerve fiber layer thickness, indicating the potential that changes in tractometric parameters could provide a complementary marker of the disease.

**Conclusions:**

This review enhances our understanding of WM changes in glaucoma and highlights the potential for dMRI tractometry as a promising tool for early detection and monitoring of the disease. By quantifying WM changes, tractometry offers valuable insights not only into the visual pathway but also into brain regions affected by glaucoma. This could lead to more accurate diagnoses, improved tracking of disease progression, and the development of targeted treatment strategies.

## 1 Introduction

Glaucoma is a leading cause of irreversible blindness (Quigley and Broman, 2006), primarily characterized by progressive optic nerve damage, retinal ganglion cell loss, and visual field deficits (Weinreb et al., 2014). Recent research has suggested that the degeneration associated with glaucoma extends beyond the retina, affecting the entire visual pathway and even some non-visual brain regions (Sun et al., 2019; Torres and Hatanaka, 2019). Thus, there is a growing need for advanced non-invasive imaging techniques to better assess brain microstructure in glaucoma patients, which could provide insights into understanding the pathophysiology of glaucoma.

Magnetic resonance imaging, a non-invasive and non-ionizing technique, has been widely used to investigate structural and functional changes in the brain in various neurological conditions, including glaucoma (Mastropasqua et al., 2015; Kang and Wan, 2022). While traditional MRI methods like T1-weighted and T2-weighted scans provide general anatomical information, advanced MRI techniques, such as dMRI, offer a deeper understanding of tissue microstructure (Alexander et al., 2019).

**Figure 1:**
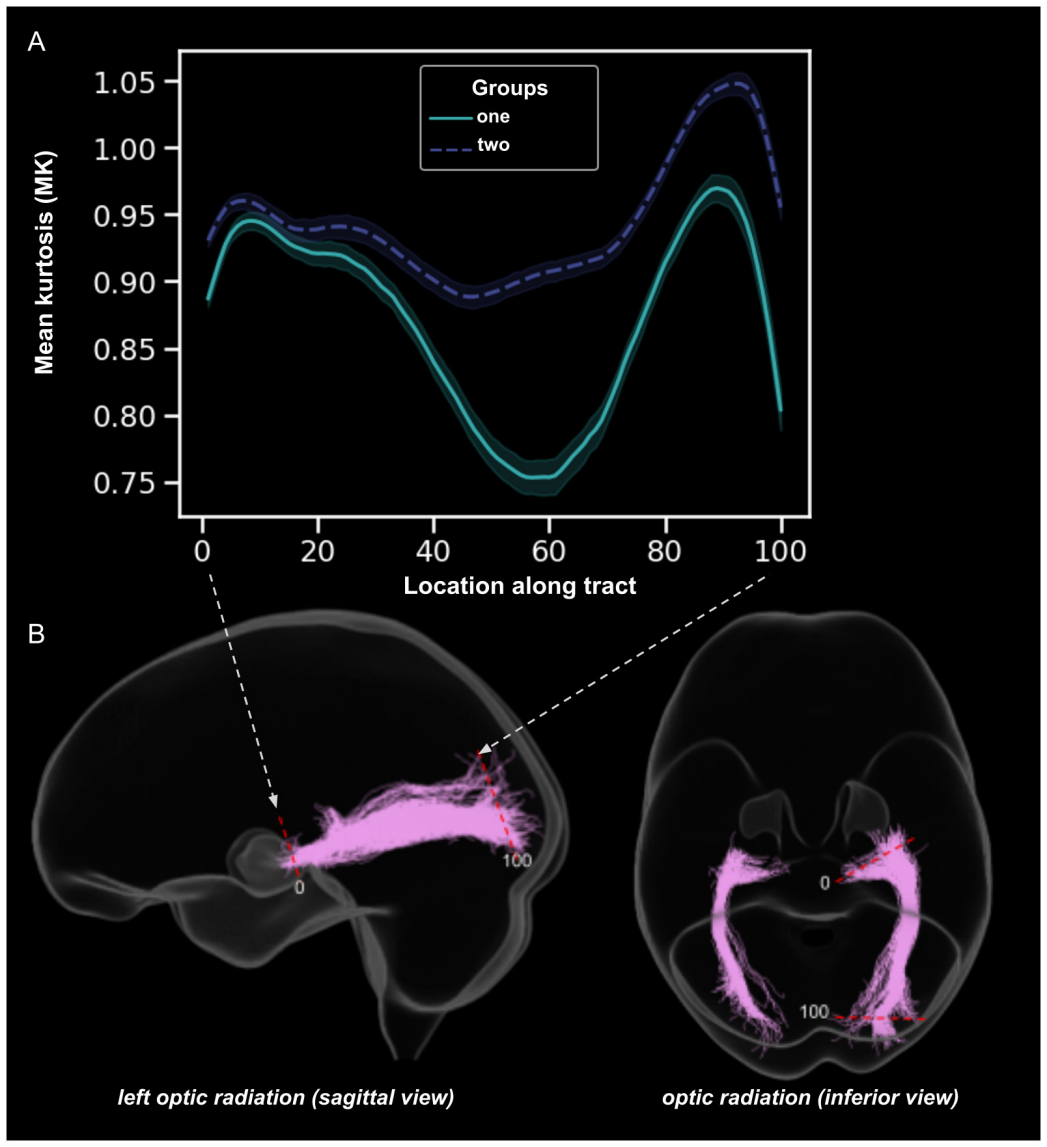
An example of tractometric analysis. It involves four main steps: tractography, tract extraction [*i.e.*, specific tracts of interest are isolated, *e.g.*, optic radiation (OR)] and cleaning (*i.e.*, removal of spurious or anatomically implausible streamlines). Once cleaned, tract quantification is performed. **(A)** Tract profiles of the left OR sampled at 100 equidistant points for group one (*e.g*., patients) and group two (*e.g*., healthy controls), with mean kurtosis (MK) a DKI parameter on the vertical axis and position along the OR on the horizontal axis. **(B)** Tractography representation of the segmented and cleaned left OR sagittal and inferior view overlaid on a glass brain. Tractometry enables detailed evaluation of microstructural properties along visual pathway white matter bundles. The figure was generated using data from a randomly selected healthy subject from the UK Biobank dataset (Sudlow et al., 2015).

Several dMRI techniques, including diffusion tensor imaging (DTI; Basser et al., 1994) diffusion kurtosis imaging (DKI; Jensen et al., 2005), neurite orientation dispersion and density imaging (NODDI; Zhang et al., 2012), and fixel-based analysis (FBA; Raffelt et al., 2015), link microscopic properties of brain tissue *in vivo* to dMRI signals revealing detailed insights into WM microstructure organization. Additionally, tractography, a computational technique that reconstructs WM tracts, has been combined with dMRI-based quantitative maps (*e.g*., mean diffusivity from DTI) in a method known as tractometry (Yeatman et al., 2012; Jeurissen et al., 2019; see Figure 1). Tractometry allows for the quantification of microstructural properties within specific WM tracts, providing valuable insights into the integrity of brain regions affected by diseases. Recent studies have applied dMRI tractometry to glaucoma research, identifying disruptions in the WM of the visual pathway and beyond, highlighting the utility of these advanced imaging techniques in understanding glaucoma's impact on the brain (Zhou et al., 2017; Miller et al., 2019).

**Figure 2:**
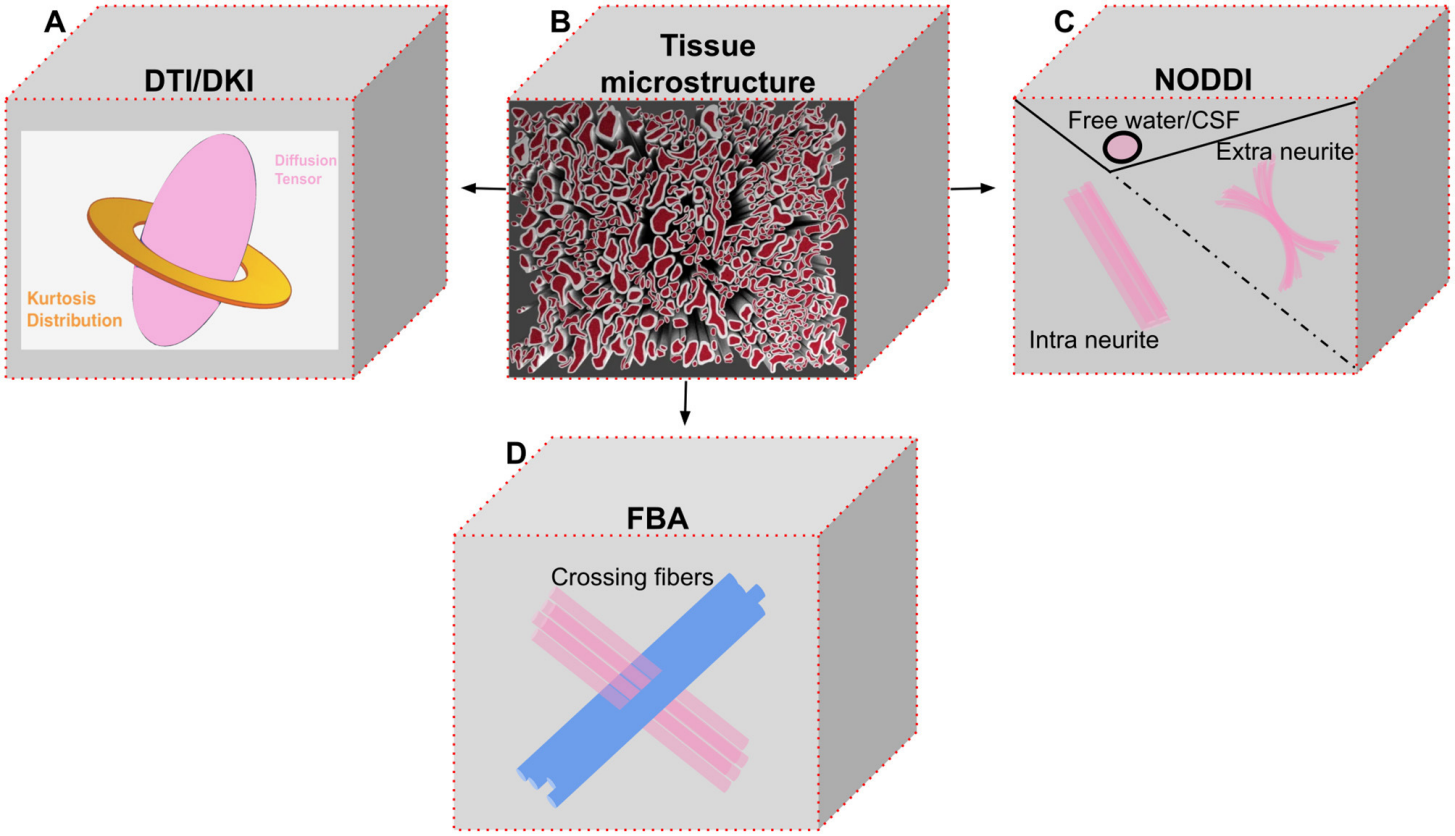
Overview of the four dMRI methods utilized in the tractometry analysis of the selected manuscripts. **(A)** Illustration of diffusion tensor imaging (DTI) and diffusion kurtosis imaging (DKI). DKI extends DTI by incorporating the kurtosis tensor to capture kurtosis distribution or non-Gaussian diffusion within the microstructural environment (Jensen and Helpern, 2010). While DTI is well-suited for quantifying voxels with a single dominant fiber orientation, DKI can also characterize more complex fiber configurations. **(B)** An example of a brain tissue microstructural environment within a voxel, different fiber configurations could be present, including single-direction fibers, crossing fibers, bending fibers, and or dispersed fibers (Tournier et al., 2011). **(C)** Neurite orientation dispersion and density imaging (NODDI) models the voxel composition by distinguishing between intra-neurite compartments (fibers aligned in the same direction), extra-neurite compartments (dispersed or bending fibers), and free water/CSF. **(D)** Fixel-based analysis (FBA) enables the identification of crossing fibers. While NODDI disentangles certain microstructural properties, such as orientational dispersion and neurite density, it does not distinguish between separate fiber populations within a voxel. Instead, it models fiber crossings as a single population with high dispersion, without separately quantifying neurite density for each population. In contrast, FBA provides more precise insights into distinct fiber pathways (Dhollander et al., 2021).

This systematic review focuses on the application of dMRI-based tractometry to study the visual pathway in glaucoma. While a general overview of dMRI methods is included for context (see Figure 2), this review emphasizes the specific role of tractometry (Figure 1), a recent approach that uses tractography for tract-specific analysis (Yeatman et al., 2012). This method aims to extract quantitative parameters along specific fiber bundles or tracts, providing insights into microstructural integrity and its relationship to glaucoma-related changes.

## 2 Materials and methods

This systematic review adhered to the PRISMA 2020 (Preferred Reporting Items for Systematic Reviews and Meta-Analyses) guidelines and checklists for reporting (Page et al., 2021).

### 2.1 Search strategy

The following databases were searched: PubMed-Medline and PubMed-Central, which contain the Cochrane Eyes and Vision Group Trials Register. Search strings were developed based on literature review and domain expertise. Title and abstract searches were conducted in the selected databases using the following terms: (glaucoma AND diffusion MRI tractometry) AND (microstructure imaging OR diffusion tensor imaging OR higher-order diffusion MRI methods OR diffusion MRI biophysical models OR diffusion-weighted imaging OR tractography). Articles published until April 11th, 2024, were included while excluding all gray literature and non-english publications. These searches were complemented by manually checking the bibliographies of all included studies.

### 2.2 Study selection

**Figure 3:**
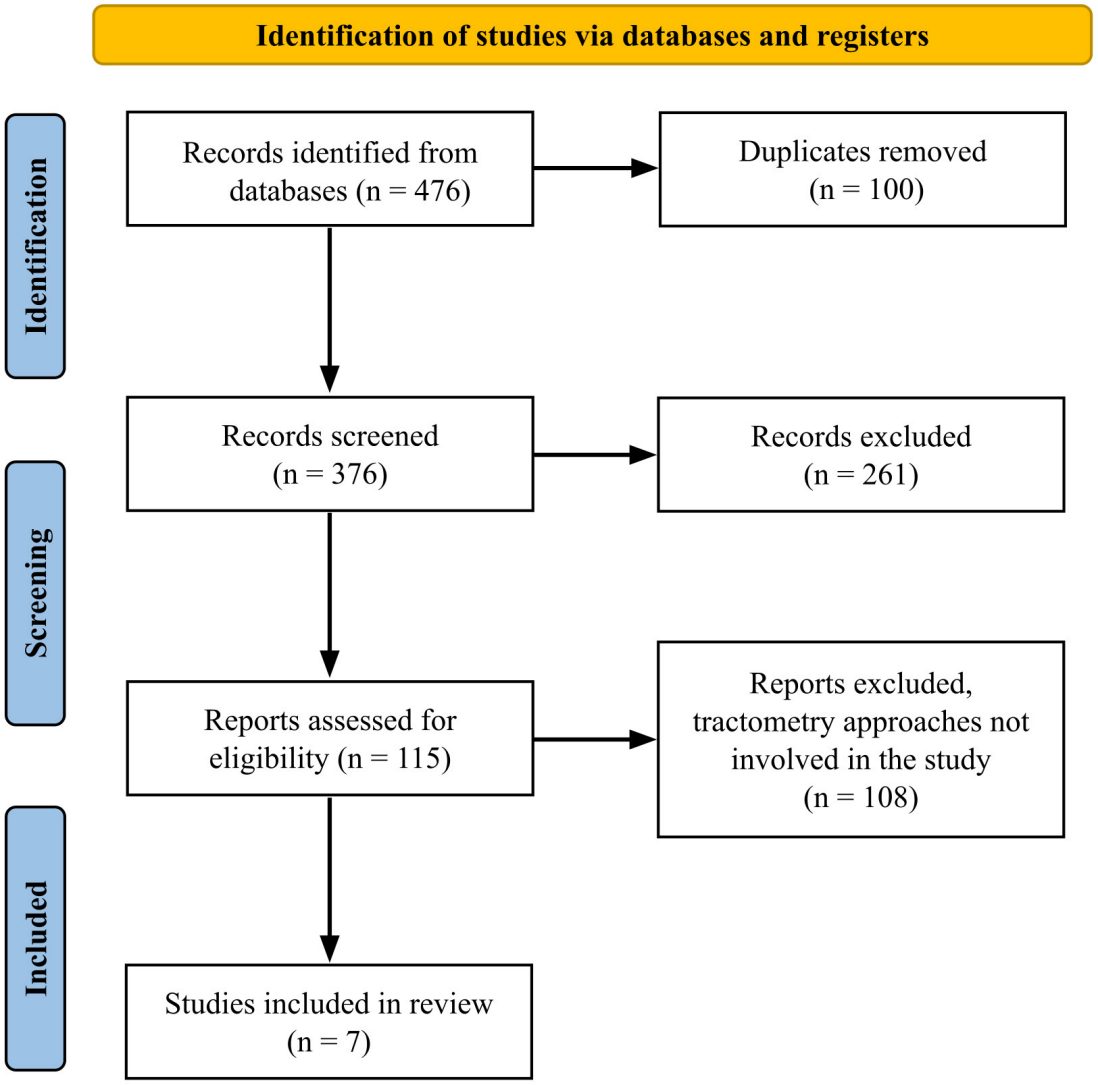
PRISMA flow diagram for study selection.

Following the PRISMA flow diagram (Figure 3), duplicates were removed using EndNote 21, and initial selection was carried out by screening titles and abstracts. After initial selection, full texts were evaluated for eligibility.

### 2.3 Study design

Cohort studies, comparative studies, cross-sectional analyses, and randomized controlled trials were included. Reviews, meta-analyses, and case studies with patients with other known neuropathologies, such as Alzheimer's and Sturge-Weber Syndrome, were excluded.

### 2.4 Participants

Selected studies included those on patients affected by any major subtype of glaucoma: primary open-angle glaucoma (POAG), primary angle closure glaucoma (PACG), normal tension glaucoma (NTG), high tension glaucoma (HTG), and neovascular glaucoma (NVG). Details on these glaucoma subtypes can be found in the following reference (Kolb et al., 1995). Glaucomatous changes could be unilateral or bilateral, and any stage of disease progression was included. All studies were selected based on their inclusion of dMRI-based tractometry to study microstructural changes (Table 1).

**Table 1:** Demographic and clinical characteristics of studies selected.

**References**	**G Type**	**N Patients**		**Age (years) Mean** ±**SD**		**Sex M/F (** ***N*** **)**		**Clinical characteristics of glaucoma subjects**
	**G**	**C**	**G**	**C**	**G**	**C**	
Tellouck et al. (2016)	NS	50	50	61.9 ± 6.9	61.9 ± 7.0	20/30	20/30	Patients were categorized into early, mild and severe groups with the HPA classification.
Zhou et al. (2017)	POAG	11	11	60.0 ± 9.2	55.9 ± 7.5	4/7	7/4	NS
Miller et al. (2019)	NS	6	6	53.3 ± 17.4	24 ± 5.3	2/4	2/4	A diagnosis of either primary open-angle, pigment dispersion, pseudoexfoliation, or chronic angle-closure glaucoma; IOPs > 22 mmHg.
Haykal et al. (2020)	NS	15	15	67.6 ± 7.4	70.1 ± 7.4	8/7	8/7	Glaucoma patients having been diagnosed with glaucoma in at least one eye were selected.
Hanekamp et al. (2021)	POAG NTG	47	41	GL1: 63.29 GL2: 51.93	HC1: 64.05 HC2: 52.43	31/16	22/19	17 POAG, 30 NTG
Ogawa et al. (2022)	POAG NTG	17	30	56.6	51.4	9/8	16/14	4 POAG, 11 NTG, 1 PEXG, 1 SOAG.
Kruper et al. (2024)	NS	905	5,292	68 ± 7	62 ± 7	NS	NS	Glaucoma in at least one eye; a dMRI data acquisition and a final visual acuity logMAR of less than or equal to 0.3 if measured (from UKBB data field 5,201)

Demographic and clinical details of glaucomatous subjects (G) and healthy controls (C) for each study included in this systematic review. GL, glaucoma group; HC, healthy control group; HPA, Hodapp-Parrish-Anderson; logMAR, log of the Minimum Angle of Resolution; NS, non-specific; NTG, normal tension glaucoma; POAG, primary open-angle glaucoma; PEXG, pseudoexfoliation glaucoma; SOAG, secondary open-angle glaucoma.

### 2.5 Data analysis

The primary outcome of this systematic review was a current understanding of the changes associated with glaucomatous effects on the brain, particularly WM within the visual pathway, as revealed by the dMRI tractometry methodology. Neuroimaging results of each of the seven studies were analyzed and presented (Table 2). The dMRI methods and their derived parameters included in this review are summarized below (Figure 2). We have also included a brief overview of the two methods of tractography that were used in the selected papers.

**Table 2:** Outcomes of studies with dMRI tractometry technique.

**References**	**Brain visual pathway area analyzed**	**Diffusion MRI, tractography methods and tractometry software used**	**MRI system and field strength**	**Parameters analyzed**	**Results in glaucoma patients relative to controls**
Tellouck et al. (2016)	Optic radiation	DTI Deterministic tractography Olea Medical software	MR750 3-Tesla scanner with a 32-channel head coil (GE Medical Systems, Milwaukee, WI, USA)	FA, MD, RD, AD. Correlation with disease severity.	Reduced FA and higher RD in OR. OR FA significantly correlated with homolateral functional and structural damage of glaucoma, RNFL thickness and vCD ratio. Volume and DTI parameters of other brain structures (including the hippocampus) were not significantly different between glaucoma patients and controls.
Zhou et al. (2017)	Optic radiation, optic tract, primary visual cortex	DTI Probabilistic tractography AFQ	Magnetom Trio, Siemens 3-Tesla scanner with a 8-channel head coil (Erlangen, Germany)	V1 area and volume. FA MD, RD and AD. Correlation with clinical measures of glaucoma.	Reduced area and volume of V1. Reduced FA and AD along OT and OR while RD increased. MD trends did not reach statistical significance. No DTI parameters correlated with RNFL thickness.
Miller et al. (2019)	Optic nerve	DTI Probabilistic tractography AFQ	MR750 3-Tesla scanner with a 32-channel head coil (GE Healthcare, Inc., Chicago, IL, USA)	FA MD, RD and AD and correlation with clinical measures of glaucoma.	Reduced FA and increased MD and RD in the ONs of eyes with advanced glaucoma. Reductions in ON FA correlated with clinical measures of glaucoma (vCD, RNFL, and VFI).
Haykal et al. (2020)	Optic nerve	DTI FBA Deterministic tractography Custom code	Magnetom Prisma 3-Tesla scanner with a 64-channel head coil (Siemens, Erlangen, Germany).	FA, MD, FD, FC and FDC.	Reduced FA in all three segments of the ON, Increased MD in ON intracranial segment. Reduced FD along the entire ON length. Reduced FC at the intraorbital and intracranial segments, but not the intracanalicular segment.
Hanekamp et al., 2021	37 major WM tracts including optic radiation	DTI Probabilistic tractography Vistasoft (Compute_FA_AlongFG)	Philips Intera 3-Tesla scanner with a 8-channel head coil (Philips, Eindhoven, The Netherlands) 3.0 T Siemens Magnetom Trio A Tim System scanner with a 32-channel head coil (Siemens, Erlangen, Germany)	FA and MD. Correlation with clinical measures of glaucoma.	Reduced FA and increased MD across all tract categories.
Ogawa et al. (2022)	Optic radiation, optic tract	DTI NODDI Probabilistic tractography AFQ	Magnetom Trio A Tim 3-Tesla scanner with a 32-channel head coil (Siemens, Erlangen, Germany)	FA, MD and MTV. ICVF, ODI, IsoV, and qT1. Correlation with VF test scores.	Reduced FA, ICVF and increased MD, RD along OR. Reduced FA, MTV and increased MD, qT1, ODI along OT. ODI showed significant correlation with VF in OT.
Kruper et al. (2024)	Optic radiation, corticospinal tract and uncinate	DKI Probabilistic tractography AFQ	3-Tesla Siemens Skyra system using a 32-channel head coil.	FA, MD, and MK.	Reduced FA and increased MD in the posterior OR. Slightly increased FA and reduced MD in the anterior OR. Lower MK in OR and increased in specific locations along CST and UNC.

AFQ, Automated Fiber Quantification; AD, axial diffusivity; BA, Brodmann area; dMRI, diffusion MRI; DWI, diffusion-weighted imaging; FA, fractional anisotropy; FBA, fixel-based analysis; FD, fiber density; FC, fiber-bundle cross section; FDC, fiber density and bundle cross section; FISO, Fraction of isotropic diffusion; GCC, ganglion cell complex; ICVF, intracellular volume fraction; IsoV, isotropic volume fraction; LGN, lateral geniculate nucleus; MD, mean diffusivity; MK, mean kurtosis; MTV, macromolecular tissue volume; NODDI, neurite orientation dispersion and density imaging; NS, non-specific; NTG, normal tension glaucoma; ODI, orientation dispersion index; OR, optic radiation; OT, optic tract; CST, corticospinal tract; UNC, uncinate; PACG, primary angle-closure glaucoma; POAG, primary open-angle glaucoma; qT1, quantitative T1; RD, radial diffusivity; RNFL, retinal nerve fiber layer; ROI, region of interest; TP, time-point; V1, primary visual cortex; vCD, vertical cup-to-disc ratio; VFI, visual field index; VMHC, voxel-mirrored homotopic connectivity; WM, white matter; WMM, white matter microstructure.

Diffusion tensor imaging quantifies water diffusion within WM fibers using several key metrics. Fractional anisotropy (FA) reflects the directional movement of water particles via diffusion and is often used to assess fiber integrity. Mean diffusivity (MD) measures the overall magnitude of diffusion and can indicate tissue damage when elevated. Axial diffusivity (AD) captures diffusion along the fibers length and is linked to axonal health, while radial diffusivity (RD) represents diffusion perpendicular to fibers and is often associated with myelin integrity (Le Bihan et al., 2001).

However, DTI assumes that water moves in a simple, unrestricted or unhindered path (Gaussian diffusion), which limits its ability to capture complex tissue structures. To address this, DKI extends DTI by capturing deviations from Gaussian diffusion. It adds kurtosis metrics which are analogous to DTI metrics—mean kurtosis (MK), axial kurtosis (AK) and radial kurtosis (RK), which provide additional sensitivity to tissue complexity and heterogeneity (Jensen et al., 2005). NODDI further advances microstructural assessment by modeling the composition of different tissue compartments. It provides the neurite density index (NDI), which indicate the density of axons and dendrites; the orientation dispersion index (ODI), which quantifies the variability in fiber orientations; and the isotropic volume fraction (IsoV), which represents the proportion of free water or cerebrospinal fluid (CSF), which may also indicate fluid accumulation or tissue loss (Zhang et al., 2012). Finally, FBA addresses crossing-fiber limitations by modeling multiple fiber orientations within a voxel. It provides biologically meaningful metrics such as fiber density (FD), which estimates how densely packed the fibers are; fiber cross-section (FC), which reflect the size or thickness of fiber bundles; and fiber density and cross-section (FDC), a combined measure that reflects both micro- and macrostructural changes (Raffelt et al., 2015). These advanced models enhance the ability to assess microstructural changes associated with glaucoma in the brain's visual pathways.

In tractometry, these diffusion model parameters are mapped onto WM tracts to quantify microstructural properties along their trajectory (see Figure 1). Tractography, the method used to reconstruct these tracts, plays a central role in this process. The two main types of tractography methods are deterministic and probabilistic. Deterministic tractography, also known as streamline tractography, assumes a single principal diffusion direction within each voxel. Starting from a seed point, it follows this dominant direction to generate continuous fiber pathways (Jones, 2008). In contrast, probabilistic tractography accounts for uncertainty in diffusion orientation by modeling a distribution of possible directions within each voxel. It generates thousands of potential pathways from a given seed point, producing a probability map of connections and providing greater sensitivity in regions with complex fiber architecture. The result is a probability distribution of connections, and by selecting an appropriate threshold to exclude less likely pathways, the most probable tracts can be identified and visualized (Jones, 2008).

## 3 Results

### 3.1 Study selection

A total of 476 articles were screened using our search strategy, with 100 removed as duplicates, 261 removed through screening of the title and abstract, and 108 excluded for not including tractometry in the results. Overall, seven studies were selected to be included in this systematic review. The PRISMA flow diagram (Figure 3) provides details of the screening process.

### 3.2 Study characteristics

A total of 1,051 patients with glaucoma and 5,445 controls were considered in this systematic review. Of the studies reviewed, one included POAG patients; two included both POAG and NTG patients, and four studies were non-specific about the type of glaucoma their study participants had been diagnosed with. One study classified the severity of glaucoma into early, mild and severe groups with the Hodapp-Parrish-Anderson (HPA) classification (Tellouck et al., 2016). Patient demographic details and clinical characteristics of the selected studies are presented in Table 1. The mean age of the patient ranges from 51 to 68 years, while the control group spans a broader range, from 24 to 70 years.

The reviewed studies mostly focused on WM tracts within the visual pathway, including the ON, OT, OR, and primary visual cortex (V1). Additionally, some studies examined other tracts, such as the uncinate fasciculus (UNC) and corticospinal tract (CST). One study analyzed a comprehensive set of 37 WM bundles, including the OR.

Regarding tractography methods, five studies employed probabilistic tractography (Descoteaux et al., 2009; Jones, 2008), while two used deterministic tractography (Descoteaux et al., 2009; Jones, 2008). For tractometry analysis (see Figure 1), four studies utilized the automated fiber quantification (AFQ; Yeatman et al., 2012) tool with DTI, DKI, and NODDI, while others used DTI and FBA with alternative software such as Vistasoft, Olea Medical and custom code. A detailed breakdown of each study, including tracts analyzed, tractography methods, and tractometry tools, is provided in Table 2.

### 3.3 Findings from DTI-based tractometric studies in glaucoma

Diffusion tensor imaging quantitative parameters (*i.e*., FA, MD, AD, and RD) has been used in six of the selected seven studies that deployed tractometry to quantify microstructure properties along the visual pathway WM (Table 2). Reduced FA and increased MD and RD were the most common findings within the visual pathway (ON, OT and OR). These results were found in POAG, NTG and NS groups (Hanekamp et al., 2021; Ogawa et al., 2022; Zhou et al., 2017; Haykal et al., 2020; Miller et al., 2019; Tellouck et al., 2016). Strong correlation between FA and retinal nerve fiber layer (RNFL) thickness including vertical cup-to-disc ratio (vCD) were also observed (Tellouck et al., 2016; Zhou et al., 2017; Miller et al., 2019). The following studies, (Hanekamp et al., 2021; Ogawa et al., 2022; Zhou et al., 2017; Haykal et al., 2020; Miller et al., 2019; Tellouck et al., 2016) observed reduced area and volume in the V1 region with elevated MD and RD while FA and AD were decreased. Furthermore (Hanekamp et al., 2021; Ogawa et al., 2022; Zhou et al., 2017; Haykal et al., 2020; Miller et al., 2019; Tellouck et al., 2016), found reduced FA and increased MD across non-vision related WM tracts, such as those in the corpus callosum: forceps minor, anterior, middle, and parietal in POAG and NTG patients.

### 3.4 Findings from DKI-based tractometric studies in glaucoma

From the selected studies, only one study used DKI-based tractometry (Kruper et al., 2024). This study examined glaucoma dMRI data in a large sample from the UK Biobank and used DKI in conjunction with deep-learning convolutional neural networks (CNNs) to model the WM tissue properties within the OR. They aimed to study the effects of glaucoma on WM tissue properties of OR and to compare the classification of glaucoma using CNNs trained on tractometry measurements of the OR, against those of non-visual brain connections. They also aimed to test if glaucomatous WM changes in the OR represent accelerated aging using the glaucoma classification CNN. They found that CNNs trained on OR tissue properties using DKI based tractometry exhibited higher accuracy in classifying subjects with glaucoma compared to those trained on tractometry outputs from non-visual brain connections. The CNN trained to classify glaucoma did no better than chance in classifying glaucomatous subjects from different age groups, and the CNN trained to perform age classification could not distinguish glaucomatous subjects from age-matched controls better than chance. These results suggest that glaucomatous features of the OR that correlate with glaucoma status differ from those correlated with normal aging processes.

### 3.5 Findings from NODDI-based tractometric studies in glaucoma

Using the dMRI model NODDI, only one study (Ogawa et al., 2022) examined WM microstructure of the visual pathway in patients with POAG and NTG. In the OT of patients, compared to controls, they found significant differences in all three NODDI metrics: lower in NDI or intracellular volume fraction (ICVF) and IsoV measurements with higher ODI. In the OR, they only found significantly lower ICVF (Ogawa et al., 2022). In addition to NODDI metrics, a quantitative method called macromolecular tissue volume (MVT; Mezer et al., 2013) was used to assess the volume, while quantitative T1 (qT1; Sereno et al., 2013) was employed to evaluate the myelin properties of the OT. The results indicate a reduction in MVT measurements, suggesting a decrease in volume, alongside an increase in qT1, which points to alterations in the myelin content of the OT. The NODDI-based tractometry findings including qT1 and MVT suggest a potential disruption in both the structural and myelin characteristics of the optic tract in glaucoma patients.

### 3.6 Findings from FBA-based tractometric studies in glaucoma

To better quantify diffusion properties in voxels with multiple fiber configuration (e.g., crossing fibers), FBA-based tractometry was used for investigating the visual pathway WM in non-specific glaucoma patients (Haykal et al., 2020). This study highlighted that FD and FDC values of the intraorbital and intracanalicular segments showed significant correlations with peripapillary RNFL thickness and visual field mean deviation (VFMD). These results indicate axonal loss and gross atrophy of the ON, and the correlation between FBA measures and clinical tests of glaucoma demonstrates the potential of FBA metrics as potential biomarkers of glaucomatous ON degeneration. Moreover, focusing on degeneration of the ON segments in glaucomatous patients with POAG, pseudoexfoliative glaucoma (PEX) and pigmentary glaucoma (PG), they found significant reductions in FD and FDC along the entirety of the ON, and a loss of FC in the intraorbital and intracranial segments of the ON. The reduction of FD and FC reflects a decrease in the microscopical intra-cellular volume of axons and macroscopic decrease of the cross-sectional size of ON, respectively.

## 4 Discussion

In this systematic review, we have examined the application of diffusion MRI based tractometry in glaucoma research, with a focus on its role in assessing neurodegeneration along the brain's visual pathway. While glaucoma has traditionally been regarded as an ophthalmic disorder characterized by optic neuropathy and visual field loss, accumulating evidence suggests that it is a multifactorial neurodegenerative disease with widespread brain involvement. Although prior studies have explored dMRI techniques in glaucoma, to the best of our knowledge, no systematic review has specifically synthesized research on tractometry as a tool for quantifying white matter changes in this context. This review highlights the potential of tractometry in advancing our understanding of glaucoma-related neurodegeneration and its implications for early detection and treatment planning.

### 4.1 Microstructural changes in the visual pathway

Tractometry studies that used dMRI data representation techniques (*i.e*., DTI and DKI) and dMRI models (*i.e*., NODDI and FBA) to quantify the underlying microstructure of the visual pathway (*i.e*., ON, OT and OR) found significant differences between glaucomatous subjects and healthy controls. The changes observed in DKI and DTI metrics—specifically, the reduction in FA and MK, along visual pathways with increases in MD and RD—may suggest microstructural disruption that allows for isotropic (free) diffusion. These differences, seen in the glaucoma patient group compared to healthy controls, could reflect axonal fiber loss and myelin degradation along the WM tracts of the visual pathway. Studies demonstrated findings indicative of glaucomatous WM degeneration at specific location along the optic nerve (Miller et al., 2019; Haykal et al., 2020), optic tract (Zhou et al., 2017; Ogawa et al., 2022), and optic radiation (Tellouck et al., 2016; Zhou et al., 2017; Hanekamp et al., 2021; Ogawa et al., 2022; Kruper et al., 2024).

Previous non-dMRI tractometric studies have also found a similar trend with DTI metrics. A first *in vivo* optic nerve study looking at proximal (near optic nerve head) and distal (orbital apex) locations found increased MD and decreased FA at the proximal site compared to distal in severe glaucoma patients (Bolacchi et al., 2012). Also, a correlational study of DTI and structural measurements of the optic nerve from three established glaucoma imaging tools reported significant correlation in MD and FA with linear cup/disc ratio (LCDR) and RNFL thickness, respectively (Nucci et al., 2012). Similar findings with DTI metrics have also been observed in animal models using analysis techniques other than tractometry. In several rat glaucoma models, investigations have documented an increase in MD and a decrease in FA in the ON and OT (Hui et al., 2007; Ho et al., 2015; Yang et al., 2018). The FA from DTI is considered as a sensitive parameter for diagnosis of glaucoma within the visual pathway WM (Li et al., 2019).

To better quantify microstructural properties in glaucoma patients, a DKI-based tractometry analysis was conducted on a larger glaucoma dataset from UK Biobank (Kruper et al., 2024). This study found lower MK along the optic radiation in glaucoma patients, a finding that is further supported by other techniques, such as region-of-interest (ROI) based analysis (Xu et al., 2018). For example, a whole brain voxel-wise DKI analysis study looking at POAG patients observed decreased MK, RK, and AK (Nucci et al., 2020). In addition, compared to conventional DTI, DKI detected early microstructural changes, especially in non-visual tracts, including those involved in cognition and motor functions (Nucci et al., 2020). Since DKI is an extension of the standard DTI method, it offers complementary insights. For example, the observed decrease in RK within the optic tract in glaucoma patients suggests that water diffusion perpendicular to the axon axis is less restricted, which aligns with the increased RD seen in DTI (Xu et al., 2018). Although DKI shows higher sensitivity and specificity in evaluating the microstructure compared to DTI, both DTI and DKI do not directly correlate with specific types of tissue properties, such as axon diameter, myelination, axonal size or axonal packing density.

Advanced dMRI models, such as NODDI and FBA, allow for a more detailed assessment of cellular tissue architecture *in vivo*, enabling non-invasive mapping of histological features. Unlike traditional DTI and DKI, which provide non-specific measures of diffusion, NODDI and FBA aim to offer greater biological specificity. Studies using NODDI-based tractometry observed significant differences in all three NODDI parameters, NDI, IsoV and ODI along optic tract and optic radiation of glaucoma subjects compared to controls (Ogawa et al., 2022). Another study looking at ON using fixel-based analysis tractometry identified a reduction in FD in the entire tract length (Haykal et al., 2020).

These findings from dMRI-based tractometry reveal a consistent relationship between the underlying microstructural changes in glaucoma patients compared to healthy controls. The observed trends across different parameters complement each other, highlighting alterations in the visual pathway. For instance, decreases in FA and increases in MD and RD along the optic nerve (ON), optic tract (OT), and optic radiation (OR) are consistent across various DTI-based tractometry studies (Hanekamp et al., 2021; Ogawa et al., 2022; Zhou et al., 2017; Haykal et al., 2020; Miller et al., 2019; Tellouck et al., 2016). Additionally, a reduction in MK within the OR, as seen in DKI-based tractometry (Kruper et al., 2024), further supports these findings. Moreover, tractometry studies using dMRI models like NODDI and FBA provide additional complementary data, showing increased ODI and decreased NDI along the OT and OR, while FD and FDC are reduced in the optic nerve (Ogawa et al., 2022; Haykal et al., 2020). Together, these results highlight the ability of tractometry in capturing microstructural disruptions in glaucoma, providing a detailed, segment-specific assessment of the disease's impact along the visual pathway.

### 4.2 Brain changes beyond the visual pathway

Two studies included in this review reported abnormalities in WM indirectly beyond the visual pathway. Hanekamp et al. (2021) identified 37 major WM tracts and assigned them to categories related to vision, cognition and motor control. They found abnormal integrity across non-vision WM tracts, including those projecting through the body of the corpus callosum (forceps minor, anterior, middle and parietal tracts; Hanekamp et al., 2021). Furthermore, subjects with glaucoma exhibited significant reductions in volume and area of V1 (Zhou et al., 2017). In addition (Tellouck et al., 2016; Zhou et al., 2017; Hanekamp et al., 2021; Ogawa et al., 2022; Kruper et al., 2024), found increased MK in specific locations along CST and UNC tracts. Non-visual diffusion alterations in glaucoma patients have been observed in other analysis techniques as well. For example, a study using structural connectivity found significantly altered WM connections between the visual cortex, the inferior/middle temporal gyrus and the parahippocampal gyrus in POAG subjects (Qu et al., 2019). Qu et al. (2019) applied areas from Brodmann atlases as nodes to construct WM tracts in the whole brain to explore changes with POAG. The mean FA of WM fiber connections were significantly decreased or increased, indicating widespread reorganization of structural connectivity in individuals with POAG compared to healthy controls. Supporting this, additional studies have demonstrated both structural and functional brain alterations in glaucoma, particularly in areas related to vision and cognition. These findings collectively suggest that glaucoma leads not only to localized visual pathway damage but also to broader neural reorganization, reflecting the brain's adaptive response to progressive visual loss (Di Ciò et al., 2020; Minosse et al., 2019; Martucci et al., 2020). The results from the dMRI tractometric analyses by Zhou et al. (2017) and Hanekamp et al. (2021), included in this review, further support the idea that glaucoma involves altered microstructural properties both within and beyond the visual pathway.

### 4.3 Therapeutic perspectives for dMRI tractometry

Several studies have correlated their findings with retinal measures, such as retinal nerve fiber layer (RNFL) thickness and visual field mean deviation (VFMD), providing strong evidence that glaucomatous damage to the retina is linked to measurable changes along the brain's visual pathways (Miller et al., 2019; Tellouck et al., 2016). The correlation between FBA and DTI-based tractometric measures of glaucomatous changes in the ON and OR, along with established clinical tests like RNFL and VFMD, underscores the potential of dMRI tractometry to provide further insights into brain changes in glaucoma. Beyond its potential diagnostic application, dMRI tractometry based on both DTI and more advanced models could offer valuable tools for evaluating therapeutic efficacy. Nucci et al. (2013) emphasized the role of DTI in detecting glaucomatous damage within the central nervous system and in tracking the impact of neuroprotective interventions over time. For instance, agents such as coenzyme Q10 (CoQ10) have demonstrated neuroprotective effects by reducing oxidative stress and supporting retinal ganglion cell survival (Martucci and Nucci, 2019; Nucci et al., 2013). Additionally, evidence of brain changes in non-visual areas (see section 4.2), including regions such as the frontal lobe and hippocampus (Frezzotti et al., 2014), highlights the broader neurodegenerative profile of glaucoma and reinforces the importance of whole-brain tractometry in evaluating disease progression and therapeutic outcomes. These findings support a paradigm shift in glaucoma management, from an exclusive focus on lowering intraocular pressure (IOP) toward integrated neuroprotective strategies, while emphasizing the need for CNS-targeted biomarkers such as advanced MRI techniques (e.g., tractometry) to monitor both disease progression and treatment efficacy (Nucci et al., 2016). In addition, integrating dMRI tractometry with clinical and functional assessments may further enhance our ability to understand and manage glaucoma as a central nervous system disorder.

### 4.4 Visual pathway tractometry in glaucoma: challenges and emerging directions

Diffusion MRI tractometry holds significant promise for advancing glaucoma diagnosis by offering detailed insights into the structural integrity of the visual pathways. However, several technical challenges remain that must be addressed to fully realize its clinical potential. One key limitation is the sensitivity of dMRI data to noise and motion artifacts, which can distort the accuracy of tractography. Additionally, the presence of multiple fiber orientations within a single voxel, such as in regions with sharp curvatures or fiber crossings, complicates the tracking process, particularly in complex brain structures like the optic radiation and Meyer's loop (Takemura et al., 2024). These challenges are further exacerbated by the proximity of intersecting pathways, such as the inferior longitudinal and fronto-occipital fasciculus, making accurate tractography even more difficult (Hofer et al., 2010).

Advancements in algorithms, such as constrained spherical deconvolution, aim to improve the resolution and accuracy of tractography, but even these methods have their limitations (Morez et al., 2021). Furthermore, the computational complexity of processing high-dimensional dMRI data and transforming it into biologically meaningful tract-specific profiles adds another layer of difficulty. The variability in imaging protocols, hardware, and software across different studies also raises concerns about the reproducibility and generalizability of findings. Artificial intelligence is increasingly being explored to address several of these limitations. Studies using deep learning models, including convolutional and recurrent neural networks, have shown promise in capturing complex spatial patterns in tractometry data (Rokem et al., 2023; Karimi and Warfield, 2024). For instance, CNNs have outperformed traditional linear models in identifying subtle alterations in the visual pathways of glaucoma patients, highlighting their potential to detect non-linear biomarkers that may be missed by conventional analyses (Rokem et al., 2023; Kruper et al., 2024). Machine learning methods are also being applied to reduce interscanner variability, enhance tractography accuracy through microstructure-informed models, and disentangle biological signals from noise using generative approaches such as autoencoders (Karimi and Warfield, 2024).

Despite these advances, challenges remain in ensuring model interpretability, generalizability to out-of-distribution data, and the availability of large-scale datasets for robust training. Moving forward, efforts should focus on developing more robust and standardized imaging protocols to reduce variability and improve the reproducibility of dMRI tractometry across different populations and research groups. Continued improvement of tractography algorithms augmented by machine learning techniques, particularly in regions with complex fiber configurations, will be crucial for enhancing the precision of visual pathway mapping in glaucoma. Additionally, large-scale multi-center studies, including longitudinal designs are needed to validate the clinical utility of dMRI tractometry as a reliable biomarker for glaucomatous degeneration. With these advancements, dMRI tractometry has the potential to aid the clinical workflow in the diagnosis and monitoring of glaucoma, providing critical insights into the disease.

### 4.5 Study limitations

This systematic review includes studies that investigated subjects with a broad range of glaucoma subtypes, each with distinct underlying causes. For example, while POAG would be most glaucoma cases, studies with unspecified glaucoma could also include a small number of individuals with angle closure glaucoma, which has a nearly distinct disease etiology. Thus, the heterogeneity among these subtypes, given their unique pathophysiological features and varying treatment responses, may confound the results and make cross-study comparisons challenging. Furthermore, most studies included participants with varying ages, disease severity, and disease duration, adding another layer of heterogeneity that could influence the outcomes.

## 5 Conclusion

This systematic review synthesizes findings from seven selected studies on advanced dMRI tractometry in glaucoma, highlighting its potential for assessing microstructural alterations along the brain's visual pathway. Unlike conventional ophthalmic measures, dMRI tractometry provides a tract-specific approach to mapping, visualizing, and quantifying white matter changes associated with glaucoma. Strong correlations between tractometry findings and clinical measures, such as visual field loss and retinal nerve fiber layer thickness, underscore its promise as a tool for detecting and monitoring brain involvement in the disease.

While dMRI tractometry offers a novel perspective on glaucoma-related WM alterations, the precise mechanisms underlying these changes and their relationship to disease severity remain unclear. Continued advancements in imaging protocols, standardization efforts, and methodological refinements are essential to fully realize its clinical utility. Several of the studies included in this review have demonstrated the feasibility of implementing dMRI tractometry in clinical settings, showing that scan acquisition and analysis can be completed within practical timeframes (~3–10 min) depending on the diffusion protocol applied using standard clinical hardware (e.g., 3T scanners). As research progresses, dMRI tractometry may contribute to earlier diagnosis, improved disease monitoring, and more targeted treatment strategies, ultimately enhancing patient care.

## Data Availability

The original contributions presented in the study are included in the article/supplementary material, further inquiries can be directed to the corresponding author.
